# Editorial: Cyanobacteria: The Green *E. coli*

**DOI:** 10.3389/fbioe.2016.00007

**Published:** 2016-02-02

**Authors:** Anne M. Ruffing, Toivo Kallas

**Affiliations:** ^1^Sandia National Laboratories, Department of Bioenergy and Defense Technologies, Albuquerque, NM, USA; ^2^Algoma Algal Biotechnology LLC, Oshkosh, WI, USA; ^3^Department of Biology, University of Wisconsin-Oshkosh, Oshkosh, WI, USA

**Keywords:** cyanobacteria, green *E. coli*, cyanobacterial metabolites, cyanobacterial biofuels, cyanobacterial products, genetic modification of cyanobacteria, genetic engineering of cyanobacteria

**The Editorial on the Research Topic**

**Cyanobacteria: The Green*E. coli***

As the world struggles to reduce its dependence on fossil fuels and curb greenhouse gas emissions, industrial biotechnology is also “going green.” *Escherichia coli* has long been used as a model Gram-negative bacterium, not only for fundamental research but also for industrial applications. Recently, however, cyanobacteria have emerged as candidate chassis for the production of commodity fuels and chemicals, using CO_2_ and sunlight as their main sources of carbon and energy (Ducat et al., 2011; Machado and Atsumi, 2012). In addition to their potential for reducing greenhouse gas emissions and lowering production costs, cyanobacteria have naturally efficient pathways for the production of carbohydrates and proteins as well as metabolites such as vitamins and carotenoids, which are of importance in the nutraceutical industry (Wang et al., 2014). The unique metabolic and regulatory pathways present in cyanobacteria present new challenges for metabolic engineers and synthetic biologists. Moreover, their requirement for light and the dynamic regulatory mechanisms of the diurnal cycle further complicate the development and application of cyanobacteria for industrial applications. Consequently, significant advancements in cyanobacterial engineering and strain development are necessary for the development of a truly “green *E. coli*.”

This Research Topic highlights cyanobacteria as organisms of emerging industrial relevance and discusses unique challenges posed by these photosynthetic hosts. Original Research articles in this issue demonstrate the production of energy-dense biofuels in engineered cyanobacterial hosts, including alkanes (Klahn et al.), free fatty acids (Ruffing; Work et al.), and terpenes (Davies et al.). The use of cyanobacteria for the production of plant secondary metabolites is reviewed by Xue and He. In addition to the production of metabolites, Jensen and Leister present their Opinion article on exploiting cyanobacteria for modifying photosynthesis, allowing for more rapid modification and characterization compared to plant hosts. Collectively, these articles provide a broad overview of the potential applications enabled by cyanobacterial hosts, yet this is certainly not an exhaustive list. Cyanobacteria have been successfully engineered in other applications including biosensing, bioremediation, protein production, and hydrogen production (Ruffing, 2011), and diverse, new applications for cyanobacteria are most certainly under development.

A common challenge in engineering cyanobacteria is often the paucity of characterized genetic tools available for cyanobacterial hosts. Oftentimes, genetic tools developed for *E. coli* may be applied in a cyanobacterial host, yet the function of these *E. coli* elements can vary greatly when expressed in a cyanobacterium (Markley et al., 2015). The synthetic constructs may also be more effective when integrated with the cyanobacterial metabolism, requiring the development of new tools that interface with the circadian rhythm of cyanobacteria and the diurnal nature of photosynthetic metabolism. This Research Topic includes a review by Camsund and Lindblad of transcriptional tools and design principles for engineering transcriptional systems in cyanobacteria. Further, Branco dos Santos et al. present the compelling perspective that mitigation of carbon emissions is more likely to succeed if cyanobacteria are viewed as cell factories for bioproducts, similar to *E. coli*, rather than solely as a means for CO_2_ capture.

In addition to genetic tool availability for cyanobacteria, other unique challenges result from the use of these photosynthetic hosts in biotechnology applications. Metabolite production, particularly biofuel synthesis, may have toxic or detrimental effects on the physiology of the cyanobacterial host, as described in an Original Research article by Pei et al., analyzing the effects of exogenous biofuels on the protein network in *Synechocystis* sp. PCC 6803. The photosynthetic constraints of light sensing and light harvesting are discussed in a Perspective by Montgomery. In his Hypothesis and Theory article, Burnap analyzes systems-level constraints of autotrophic productivity in cyanobacteria and argues that intracellular crowding imposes limitations on the allocation of proteomic resources. An interesting case-in-point may be the recent rediscovery of a fast-growing cyanobacterium, with a generation time of ~2 h that apparently does so in part by not devoting resources to glycogen synthesis during early stages of growth (Yu et al., 2015). Lastly, Jones provides an important Perspective on a largely ignored problem in cyanobacterial engineering, namely that of genetic instability. As illustrated by the articles in this Research Topic, the successes and challenges of developing cyanobacteria as cell factories make this an exciting time for the green *E. coli*.

## Author Contributions

Dr. AR and Prof. TK served as co-editors for the Research Topic: Cyanobacteria: The Green *E. coli*. Dr. AR conceived of the idea for the research topic. Both Dr. AR and Prof. TK served as editors for the manuscripts in this Research Topic and contributed to writing the introductory editorial.

## Conflict of Interest Statement

The authors declare that the research was conducted in the absence of any commercial or financial relationships that could be construed as a potential conflict of interest.
